# Neuroprotective effects of *Magnoliae Flos* extract in mouse hippocampal neuronal cells

**DOI:** 10.1038/s41598-018-28055-z

**Published:** 2018-06-26

**Authors:** Youn Sik Jung, Jin Bae Weon, Woo Seung Yang, Gahee Ryu, Choong Je Ma

**Affiliations:** 1 Department of Medical Biomaterials Engineering, College of Biomedical science, Kangwon National University, Chuncheon, 200-701 Korea; 2 Institute of Bioscience and Biotechnology, Kangwon National University, Chuncheon, 200-701 Korea

## Abstract

*Magnoliae Flos* (MF) is a traditional medicinal herb used for managing rhinitis, sinusitis and headache. The purpose of the present study was to determine the neuroprotective effect of MF against glutamate-induced oxidative stress and to assess the underlying mechanism. Glutamate is a major endogenous excitatory neurotransmitter in the brain and contributes to the development of neurodegenerative diseases by excessive activation. MF extract was subjected to a neuroprotective effect assay in HT22 mouse hippocampal cells. The mechanism underlying the neuroprotective effect of MF extract was evaluated by assaying reactive oxygen species (ROS) levels, intracellular Ca^2+^ levels, mitochondrial membrane potential, glutathione level and antioxidant enzyme activity in HT22 cells. MF extract significantly decreased glutamate-induced death of HT22 cells (80.83 ± 7.34% relative neuroprotection). MF extract reduced the intracellular ROS and Ca^2+^ levels and increased the glutathione level and glutathione reductase and glutathione peroxide activities. Moreover, MF extract attenuated the mitochondrial membrane potential in HT22 cells. These results suggested that MF extract exerts a neuroprotective effect against oxidative stress HT22 cells, which was mediated by its antioxidant activity.

## Introduction

The *Magnoliae Flos* (MF) is the dried flower buds of *Magnolia denudata* or related species and a botanical drug officially listed in the Pharmacopoeia of Asian Countries. The drug is known as Shin-Yi in Korea. The major components of MF are α-pinene, cineole, (−) coclaurine, and (+)-reticuline. The herbal drug has been used for managing nasal congestion with headache, sinusitis and allergic rhinitis^1^. It has a wide range of pharmacological effects, including antirheumatic, antiangiogenic, antiallergic, anti-inflammatory, and antimicrobial activities^2^.

Glutamate is an endogenous excitatory neurotransmitter in the central nervous system (CNS). It plays important roles in the synapse in memory, neuronal differentiation, and neural transmission^3^. However, a high concentration of glutamate leads to loss of learning and memory and is involved in the pathogenesis of neurodegenerative disorders, including Alzheimer’s disease (AD) and Parkinson’s disease (PD)^4^. Two common pathways of glutamate neurotoxicity have been described. One is the excitotoxic pathway, which is mediated by ionotropic glutamate receptors. The mechanism of excitotoxicity has been extensively characterized, and it is believed that transient Ca^2+^ fluxes lead to alterations in calcium homeostasis, increases in the levels of reactive oxygen species (ROS), and ultimately cell death^5^. The second distinct pathway in glutamate toxicity does not involve glutamate receptors, but rather a glutamate/cystine antiporter, which is required for the delivery of cystine into neuronal cells^6^. Inhibition of cystine uptake by high concentrations of extracellular glutamate leads to an imbalance in cellular cysteine homeostasis, a reduction in cellular glutathione levels, and the accumulation of ROS. Glutamate-induced oxidative toxicity has been described in neuronal cell lines, primary neuronal cultures, and oligodendrocytes. This oxidative neuronal death pathway is thought to contribute to neuronal injury and degeneration in many brain disorders^7,8^. HT22 cells, an immortalized mouse hippocampal cell line, have been widely used as an *in vitro* model for elucidating the mechanism of oxidative stress-induced neurotoxicity^9^. HT22 cells lack a functional ionotropic glutamate receptor, thus excluding excitotoxicity as a cause of glutamate-triggered cell death^10^. MF also has anti-inflammatory effects against lipopolysaccharide (LPS)-induced production of NF-κB–dependent inducible nitric oxide synthase (iNOS) and pro-inflammatory cytokines in Raw264.7 murine macrophages^11^. However, MF has not been reported to be a neuroprotectant that can reduce glutamate-induced apoptosis. Therefore, we examined the protective effect of MF against glutamate-induced oxidative stress in a mouse hippocampal neuronal cell line.

## Materials and Methods

### Plant materials

*Magnoliae Flos* was purchased from Kyungdongmart (Seoul, Korea) and authenticated by Dr. Young Bae Seo, a Professor at the College of Oriental Medicine, Daejeon University (Daejeon, Korea). Voucher specimens were deposited at Kangwon National University in Chuncheon, Korea (no. CJ080M). The steams of MF (600 g) were extracted with 80% methanol three times for 90 min each by ultrasonication. The extract (13.66 g) was evaporated and freeze-dried to a powder.

### Cell culture

HT22 cells, a neuronal cell line derived from the mouse hippocampus, are used to study the mechanisms of glutamate-induced cell death. HT22 cells were cultured in DMEM supplemented with 10% (v/v) fetal bovine serum (FBS), 1% penicillin/streptomycin, NaHCO_3_ (2 mg/mL), and 15 mM HEPES and were maintained at 37 °C in a humidified atmosphere containing 5% CO_2_.

### Cell viability

Cell viability was determined by MTT assay as described previously^12^. Cultured HT22 cells were seed at 1.7 × 10^4^ /well in 48-well plates and incubated for 24 h at 37 °C in 5% CO_2_. Cells were treated with 1, 10, and 100 µg/ml MF and 50 µM Trolox (water-soluble analog of vitamin E sold: positive control) for 1 h, glutamate (2 mM) was added and cells incubated for 24 h. After incubation, 1 mg/mL MTT solution was added to each well for 3 h. MTT formazan was dissolved by dimethyl sulfoxide (DMSO) and the optical density at 570 nm was measured using an enzyme-linked immunosorbent assay (ELISA) reader.

### Measurement of intracellular ROS levels

ROS formation induces neuronal cell death due to oxidative stress. Glutamate is involved in ROS production through NMDA receptor activation and intercellular Ca^2+^ accumulation. We evaluated ROS production using the dye 2′7′-dichlorofluorescein diacetate (DCF-DA) in HT22 cells. Three concentrations of ENS sample were treated with 2 mM glutamate for 8 h in the seeding cells. Subsequently, 10 µM DCF-DA was added to the cells, followed by incubation at 37 °C for 30 min. DMEM was removed, cells washed twice with phosphate-buffered saline (PBS; 0.01 M, pH 7.4) and extracted with 1% Triton X-100 in PBS (0.01 M, pH 7.4) for 10 min at 37 °C. Fluorescence was measured at an excitation wavelength of 490 nm and emission wavelength of 525 nm for detection of ROS.

### Measurement of Ca^2+^ levels

High concentrations of glutamate lead to intercellular Ca^2+^ accumulation due to activation of NMDA receptors. Increased Ca^2+^ levels have been implicated in neuronal cell death through depolarization of the mitochondrial membrane. The cytosolic Ca^2+^ concentration in HT22 cells was measured using Fura-2AM. HT22 cells were cultured at 37 °C in 5% CO_2_. HT22 cells were then treated with MF (1,10 and 100 *µ*g/mL), 2 *µ*M Fura-AM, and glutamate. After 20 min, cells were washed with HEPES buffer saline and incubated for 1 h. Fluorescence was monitored at an excitation wavelength of 380 nm with fixed emission at 510 nm.

### Measurement of mitochondrial membrane potential (MMP)

MMP was measured using the fluorescent dye Rh123, as reported previously^13^. Rh123 was added to HT22 cells to a final concentration of 10 µM for 30 min at 37 °C after the cells had been treated and washed with PBS. Fluorescence was monitored at an excitation wavelength of 488 nm with fixed emission at 525 nm.

### Measurement of total glutathione content and antioxidant enzyme activities

Cells were seeded onto six-well plates at a density of 2 × 10^4^/ml and incubated for 24 h. Cells were treated with test compounds for 1 h, followed by 2 mM glutamate. After 24 h, cells were washed twice with PBS. The cell lysate was centrifuged for 30 min at 10,000 g at 4 °C, and the supernatant was used for measurements of antioxidant activity and GSH contents.

### Free-radical scavenging assay

A 1,1-diphenyl-2-picrylhydrazyl (DPPH) radical scavenging assay was conducted to evaluate antioxidant activity. Various concentrations of samples were added to 150 *µ*L of 0.4 mM DPPH methanol solution in 96-well plates. The absorbance of DPPH solution at 517 nm was measured using an ELISA reader. DPPH radical-scavenging activity was expressed and calculated as % inhibition = (1−A*s*/A*o*) × 100, where “A*s*” is sample absorbance and “A*o*” is absorbance of only DPPH solution.

### Statistics

All results are expressed as means ± S.E.M. The results were analyzed by one-way analysis of variance (ANOVA), followed by Tukey’s post hoc test. Differences were considered statistically significant at p < 0.05, 0.01 and 0.001.

## Results

### Neuroprotective effect of MF on glutamate-induced oxidative cytotoxicity

**Figure 1:**
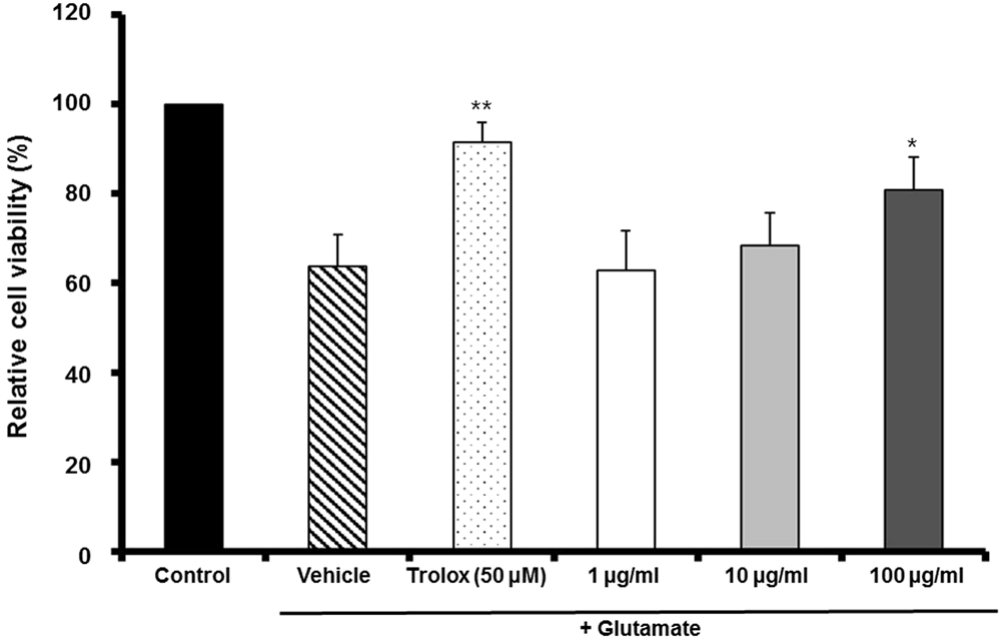
The neuroprotective effect of MF extract on glutamate-induced cell death in neuronal HT22 cells. Data represents the mean cell viability (%) ± SD of three independent experiments. *p < 0.05 and **p < 0.01 vs. glutamate-treated cells.

MTT assay was performed to investigate the neuroprotective effect of MF on glutamate-induced death of HT22 cells. We found that 2 mM glutamate treatment reduced cell viability. MF significantly improved the glutamate-mediated decrease in cell viability in Fig. 1. MF (100 *µ*g/ml) recovered cell viability to 80.83 ± 7.34% of the control, whereas the viability of the 2 mM glutamate-treated group decreased to 63.77 ± 7.11%. Trolox, as a positive control, also significantly protected HT22 cells against glutamate-induced cytotoxicity.

### MF inhibited glutamate-induced ROS accumulation

**Figure 2:**
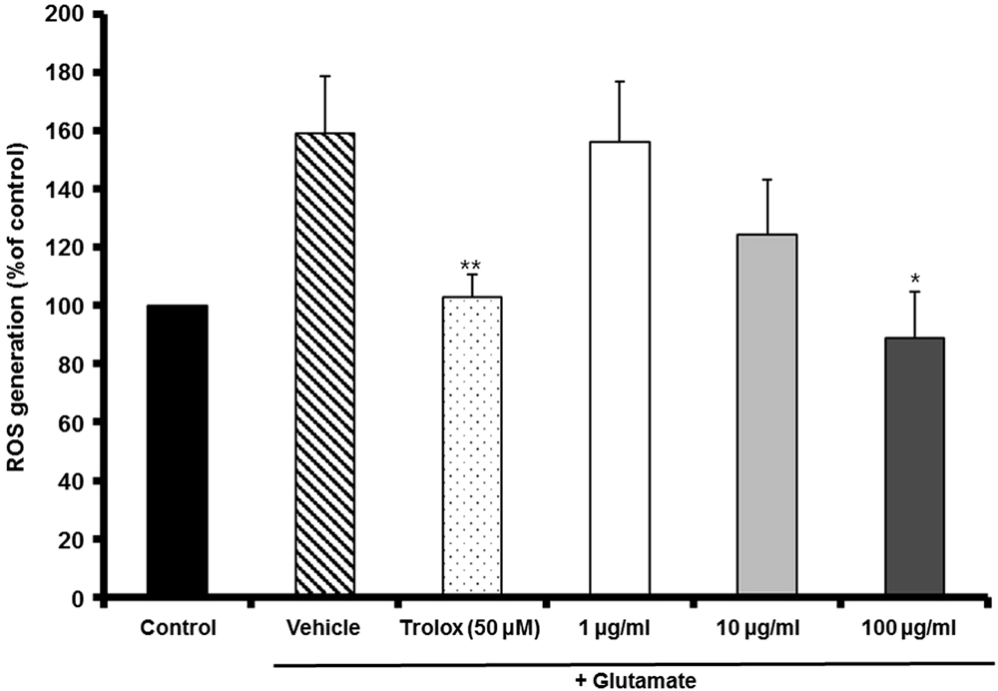
The effect of MF extract on glutamate-induced ROS generation in HT22 cells. Results are expressed as percentages of the values of control cells. *p < 0.05 and **p < 0.01 vs. glutamate-treated cells.

Glutamate-treated cells exhibited increased fluorescence compared to the controls. MF pretreatment of cells inhibited ROS overproduction in a dose-dependent manner; 100 *µ*g/mL MF significantly decreased ROS production to 88.98 ± 15.58% in Fig. 2. Therefore, MF protected HT22 cells against glutamate-induced death by inhibiting ROS production.

### MF inhibited glutamate-induced Ca^2+^ influx

**Figure 3:**
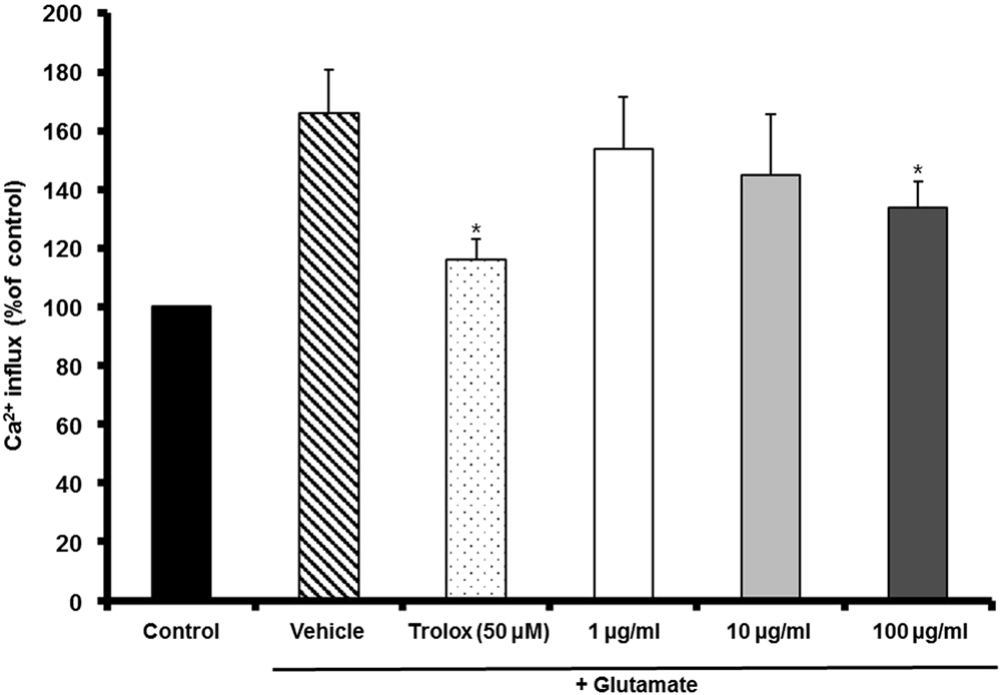
The effect of MF extract on glutamate-induced Ca^2+^ influx in HT22 cells. Results are expressed as percentages of the values of control cells. *p < 0.05 vs. glutamate-treated cells.

We evaluated the effect of MF on intracellular Ca^2+^ levels in HT22 cells using Fura-AM. As shown in Fig. 3, the intracellular Ca^2+^ concentration was increased in cells treated with glutamate to 166.07 ± 14.76%. However, the Ca^2+^ concentration in cells pretreated with MF (100 *µ*g/mL) decreased significantly compared with glutamate-treated cells (133.78 ± 9.03%).

### Inhibition by MF of glutamate-induced mitochondrial dysfunction

**Figure 4:**
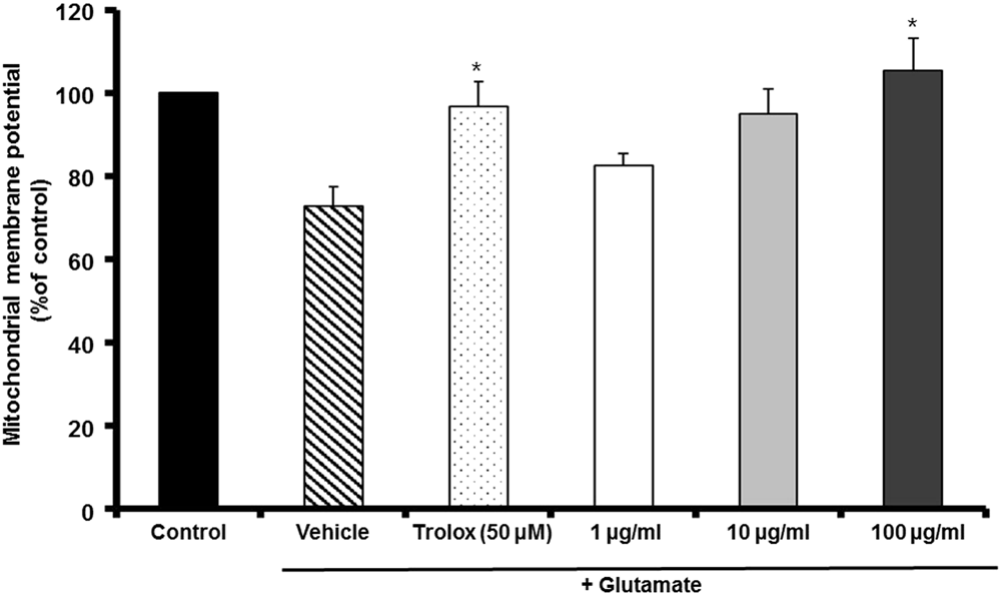
The effect of MF extract on glutamate-induced disruption of mitochondrial membrane potential in HT22 cells. Results are expressed as percentages of the values of control cells. *p < 0.05 vs. glutamate-treated cells.

To characterize the effects of Drp-1 on mitochondrial dysfunction induced by glutamate treatment, the MMP was monitored using the Rh123 probe, and the results indicated a glutamate-induced loss of MMP. ROS production causes mitochondrial injury and disrupts the MMP. Figure 4 shows that MF extract significantly recovered the mitochondrial membrane potential to 110.55 ± 10.38% of the control at 100 µg/mL.

### MF restored GSH, GR and GPx activities

**Figure 5:**
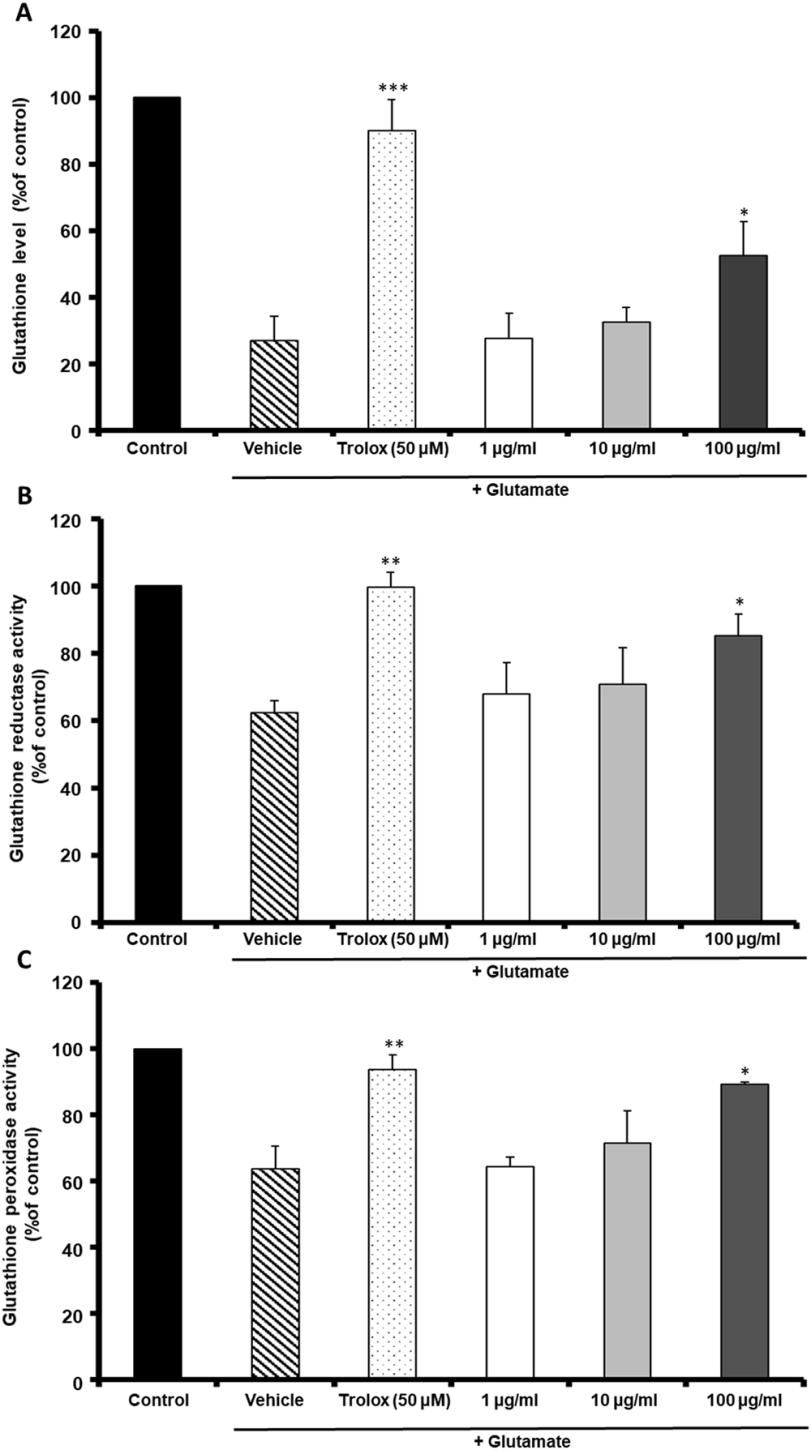
The effect of MF extract on glutathione (GSH) (**A**), glutathione reductase (GR) (**B**) and glutathione peroxidase (GPx) (**C**) activities in HT22 cells. Results are expressed as percentages of the values of control cells. *p < 0.05, **p < 0.01 and ***p < 0.001 vs. glutamate-treated cells.

We confirmed that glutamate-induced death of HT22 cells was related to oxidative stress and the treatment with MF recovered the oxidative stress condition. GSH is an important antioxidant in the CNS and glutathione reductase (GR) and glutathione peroxide (GPx) are a critical enzyme for the production of GSH. A high concentration of glutamate leads to deprivation of GSH by inhibiting cysteine uptake into cells. Depletion of GSH or antioxidant enzymes, such as GR and GPx, causes neuronal cell death. We investigated the effect of MF on GSH, GR and GPx expression levels were similar, as shown in Fig. 5. The GSH, GPx and GR expression levels in glutamate-injured cells decreased to 83.68 ± 3.18%, 71.32 ± 3.17% and 63.62 ± 6.95%, respectively. However, MF prevented the glutamate-induced depletion of GSH (92.31 ± 9.77% at 100 μg/ml), GR (81.52 ± 1.36% at 100 μg/ml) and GPx (89.26 ± 0.66%) at 100 μg/ml). These results suggest that MF exerted a neuroprotective effect, which was mediated by its antioxidant activity.

### Antioxidant activity of MF on DPPH radical scavenging assay

DPPH radical-scavenging activity was investigated to determine the antioxidant activity of MF. The DPPH radical-scavenging activity of MF showed an IC_50_ value of 437.13 mg/ml.

Positive control, trolox showed an IC_50_ value of 27.73 μg/ml. This result that MF showed antioxidant effect, but lower effect than antioxidant effect of trolox.

## Discussion

Oxidative stress elicited by glutamate induces both apoptotic and necrotic death of HT22 cells^14^. Glutamate-mediated neuronal cell death is closely associated with oxidative stress, including excessive reactive oxygen species production. Glutamate toxicity is induced mainly by glutamate receptor-mediated excitotoxicity and ROS-mediated oxidation in neuronal cells^15^. Pretreatment with an 80% methanol extract of MF exerted protective effects, reducing neuronal death at higher concentration in HT22 cells according to our results.

Antioxidant system imbalance induces Ca^2+^ influx, ROS accumulation, and lipid peroxidation. Ca^2+^ is an intracellular second messenger in the central nervous system^16^.

Increased intracellular ROS levels due to massive Ca^2+^ influx disturb the activity of lipoxygenases (LOXs), and induce lipid peroxidation and mitochondrial dysfunction, resulting in programmed cell death^17^.

GSH is an important neuronal antioxidant in biological systems that prevents and repairs peroxidative damage to lipids, proteins and nucleic acids. Loss of intracellular GSH is believed to contribute to brain aging and neurodegenerative disorders as reduced neuronal GSH levels are found in AD, PD and other neurodegenerative disorders^18^.

Disruption of mitochondrial membrane potential (ΔΨm) induced by oxidative stress is regulated by various anti- and pro-apoptotic proteins, such as Bcl-2, Bid, and Bax^19,20^.

The pathogenesis of neurodegenerative disorders is induced by multiple factors, such as Ca^2+^ overload, ROS production, lipid peroxidation, mitochondrial dysfunction, and MAPKs activation. Therefore, multi-target strategies are necessary to identify drug leads for neuroprotection or treatment of dementia.

MF extract decreased ROS generation and Ca^2+^ influx and increased GSH, GR and GPx activity in HT22 cells. Moreover, MF extract ameliorated mitochondrial dysfunction and showed DPPH radical-scavenging activity.

Our findings suggest that MF has a potent neuroprotective effect against glutamate-mediated neuronal cell death, which was associated with an antioxidant effect. MF could be valuable in the multiple-injury neuronal model, and useful for discovery of drugs effective against neurodegenerative disease.
